# Cefonicid Benzathine Salt: A Convenient, Lean, and High-Performance Protocol to Make an Old Cephalosporin Shine

**DOI:** 10.3390/antibiotics11081095

**Published:** 2022-08-12

**Authors:** Marziale Comito, Riccardo Monguzzi, Silvia Tagliapietra, Giovanni Palmisano, Giancarlo Cravotto

**Affiliations:** 1 Dipartimento di Scienza e Tecnologia del Farmaco, University of Turin, Via Pietro Giuria 9, 10125 Turin, Italy; 2 Research and Development, ACS Dobfar SpA, Via Paullo 9, 20067 Tribiano, Italy; 3 Dipartimento di Scienza e Alta Tecnologia, University of Insubria, Via Valleggio 9, 22100 Como, Italy

**Keywords:** cephalosporin, API synthesis, *N*′,*N*″-dibenzylethylene diamine diacetate, process chemistry, deformylation, amidation, sustainability

## Abstract

Cefonicid is a second-generation cephalosporin sold under the brand name Sintocef™. It is an injectable drug obtained via a freeze-drying process and is also available for oral preparations. The high-quality standard required is very challenging to satisfy, and current production protocols are characterized by steps that are lengthy and cumbersome, making the product unattractive for the international market. Industrial R&D is constantly working on the process optimization for API synthesis, with the aim of increasing productivity and decreasing production costs and waste. We herein report a new and efficient method for the synthesis of the cefonicid benzathine salt that provides a good yield and high product stability. The double-nucleophilic and lipophilic nature of *N*′,*N*″-dibenzylethylene diacetate enables the deformylation of the OH-protected group on the mandelic moiety and also enables product crystallization to occur. We demonstrate that the formyl group in the peculiar position has high reactivity, promoting an amidation reaction that deprotects a hydroxy group and generates a new C-N bond in the reaction by-product. Several amines and OH-protected groups have been studied, but none were able to replicate the excellent results of benzathine diacetate.

## 1. Introduction

The discovery and development of antibiotics, combined with their therapeutic use against many bacterial illnesses, can be considered one of humankind’s great breakthroughs, so much so that it was cited, in 2013, as one of nine ways that chemistry has changed the world [1], underlining the utmost importance of these drugs.

Although we have observed a drastic fall in the approval of new antibacterials since the 1990s [2], the market has rewarded this therapeutic class, placing it in fifth place in sales rankings in 2018, with USD 40.6 billion being sold, putting it on a par with vaccines [3]. Of the various antibiotic types available, β-lactams are the largest class in terms of production volume and market size, with USD 27.1 billion being sold in 2018 and a growth perspective of USD 34.2 billion by 2028 [4].

While penicillins and cephalosporins have been and continue to be the main β-lactam scaffolds, patients can fight infections caused by bacteria with other β-lactam subgroups, such as carbapenems, penems, monobactams, oxacephems, and carbacephems, that were discovered during the “golden age” of antibiotics in the 1960s, 1970s, and 1980s [5].

Over the last twenty years, except for the approval of fourth- and fifth-generation cephalosporins, ceftaroline fosamil [6], ceftolozane [7], ceftobiprole [8], and cefiderocol [9], the trend in pharmaceutical industry R&D has been to dredge up older cephalosporins and carbapenems and combine them with β-lactamase inhibitors to create more powerful medical weapons. Meropenem/vaborbactam, sold under the brand name Vabomere™ [10], imipenem/cilastatin/relebactam (Recarbrio™) [11], and ceftazidime with avibactam (Avycaz™) [12] constitute the recent FDA approvals in the antibacterial field.

In this landscape, where new molecular entities (NMEs) are dwindling, the possibility of a cephalosporin renaissance is realistic. In this work, we have revisited the synthetic pathway of cefonicid, one of the first cephalosporins discovered, with the aim of making it appealing to new pharmaceutical research opportunities. Cefonicid is a common, second-generation cephalosporin that was patented in 1978 [13] by Glaxo Wellcome (now GlaxoSmithkline). It is parenterally administered and used for urinary tract infections, lower respiratory tract infections, and soft tissue and bone infections [14]. Although its long half-life (4.6 h) and cost-effective, once-daily dosage regimen are the major pharmacokinetic advantages found in this generation of cephalosporins [15,16], its marketplace is currently restricted to low- and middle-income countries (LMICs), and its appeal has fallen even lower.

**Figure 1:**
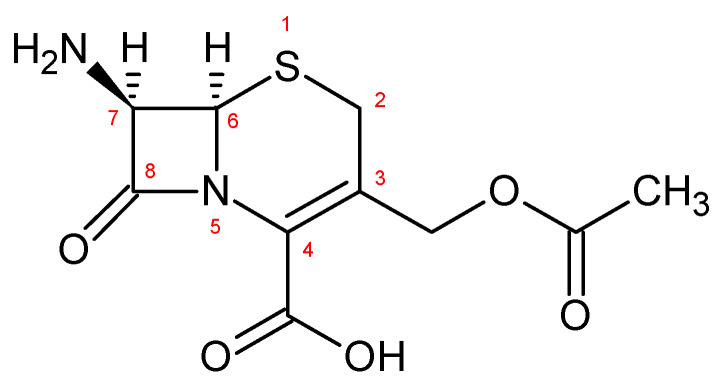
7-ACA structure.

Chemically, the general concept behind producing these semi-synthetic molecules involves derivatization at the 3- and 7- positions of 7-aminocephalosporanic acid (7-ACA), which is the backbone of almost all cephalosporin-based antibiotics and is obtained via a modern and environmentally friendly biocatalytic process from cephalosporin C [5,17,18], as designed in Figure 1.

In the specific case of cefonicid, the presence of the sulfonic acid moiety on the 1-Me tetrazole ring dramatically enhances solubility in water. Industrially, this high affinity for water explains why freeze-drying is a sterile process for the injectable form, and consequently, why even cumbersome and lengthy synthetic steps, followed by purification with chromatography or ion-exchange resins, characterized the first synthetic protocols [19,20,21]. Obtaining stable and high-purity grades of an oral drug before the lyophilization step has been the main goal of all synthetic pathways developed over the years, while at the same time, being the biggest challenge to face for process industrialization.

We have very recently reported a simple, energy-saving, and cost-effective protocol that is also scalable for commercial production for the synthesis of the key cefonicid intermediate, 7-SACA (**4**) [22]. This intermediate, with a free amino group in the 7-position, was formed in an S_N_1 reaction using a BF_3_ complex in acetonitrile as the Lewis acid, starting from 7-ACA and an S-nucleophile scaffold, which played the activator role for the acetoxy group in the 3-position and provided a reduction in waste.

In pursuit of the challenging goal of improving the total synthesis of cefonicid, and therefore, ensuring a more profitable production, we retrieved an old, synthetic pathway and designed and developed a relevant improvement by focusing our attention on the amidation of the 7-position and OH deprotection. The greatest challenge facing process chemistry is that of producing the required molecules in a controlled fashion, with reproducible impurity profiles, in an economic and scalable way. The rigorous inspection of batch impurity profiles over the last few years discourages any changes or adjustments in validated processes. However, the fact that the critical E-factors (environmental-factor) of the pharma industry are estimated to be in the 25 to 100 range (kg of waste per kg of drug molecule), the pharmaceutical–chemical community has recognized the urgent need for more sustainable manufacturing via the design of cost-effective and greener synthetic routes [23,24,25].

This situation has encouraged us to develop a new, synthetic path for the abovementioned cephalosporin, starting from the restrictions imposed by its chemical behavior and turning them into the driving force for a sustainable route to highly efficient production. Considering the old chemistry of cefonicid preparation, the conversion to modern, sustainable processes is a challenging task. We believe that this work may be pioneering in the reevaluation of cefonicid as part of the booming, β-lactam antibiotics panorama, therefore increasing its attractiveness for use in new clinicals studies in combination with β-lactamase inhibitors to fight the infinitive war against bacterial resistance.

## 2. Results and Discussion

We have investigated three different synthetic paths for the amidation of the 7-position.

### 2.1. (R)-5-Phenyl-1,3-Dioxolane-2,4-Dione

**Figure 2:**
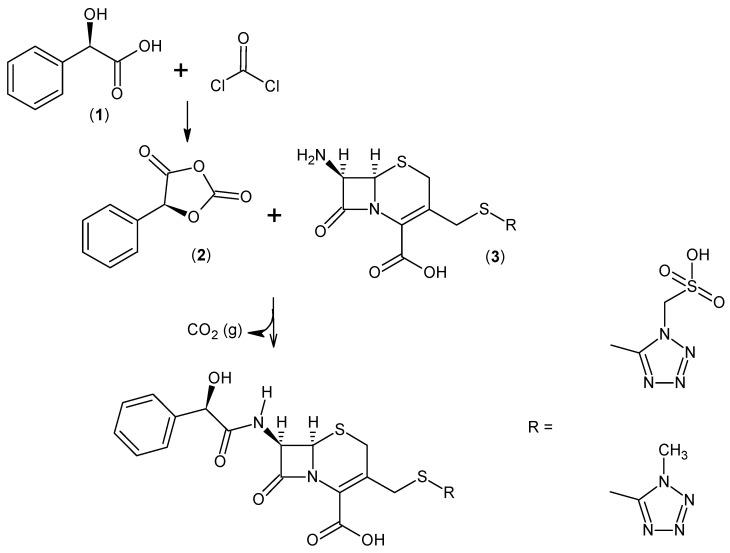
Amidation of OCA (**2**) from (*R*)-mandelic acid (**1**) and 7-SACA or 7-TACA intermediate (**3**).

*O*-carboxyanhydride (OCA) compounds are a class of five-membered rings that is widely applied in the polymeric, peptidic, and pharmaceutical fields, thanks to the ease with which the desired epimer form is generated [26,27,28,29,30,31]. The OCA subgroup from (*R*)-mandelic acid (**1**) has been the only possible way to directly access the optically active form of the 3-methylthio-tetrazolyl-7-mandeloyl cephalosporanic derivatives (cefonicid and cefamandole) from the starting substrates, and thus, bypass the hydroxy-protection–deprotection steps and the carboxylic acid activation on the mandelic moiety, as depicted in Figure 2 [32,33,34,35,36,37].

The synthesis of this compound in β-lactam manufacturing (**2**), from phosgene or its derivatives (di- and triphosgene) [38,39,40,41,42,43,44], has always been limited by safety and environmental concerns. In addition, the racemization caused by the hydrochloric acid that is released during mandelic cyclization means that several purification steps must be performed if the *R* form is to be obtained, making the process poorly convenient.

**Figure 3:**
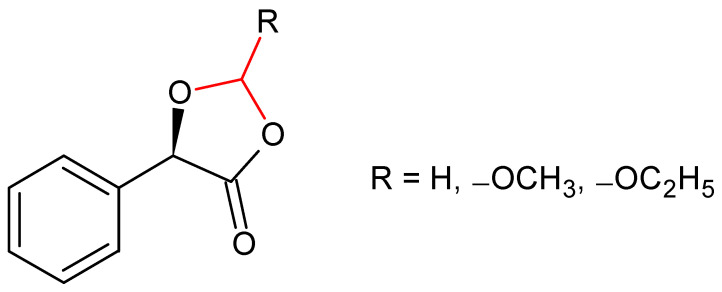
DOX, methyl-DOX, and ethyl-DOX.

Over the last few years, a new family of dioxolane compounds have emerged in the fields of biocompatible and biodegradable polymers for drug delivery in tissue engineering and food packaging, as they are easily prepared from inexpensive and sustainable feedstocks. Both 5-phenyl-1,3-dioxolane-4-one (DOX) and alkyl-DOX are part of this group, and their simple, synthetic accessibility has been proven by the fact that fewer toxic resources were used as ring-closing agents for mandelic acid (**1**) in acetone or cyclohexane, giving good isolated yields. Having observed this new frontier of compounds, we have investigated the possibility of replacing phosgene-derived OCA with DOX, which was obtained from paraformaldehyde, and with methyl- and ethyl-DOX, which were synthetized from trimethyl and triethyl orthoformate (TMOF and TEOF) [45,46,47,48,49,50], as illustrated in Figure 3.

**Figure 4:**
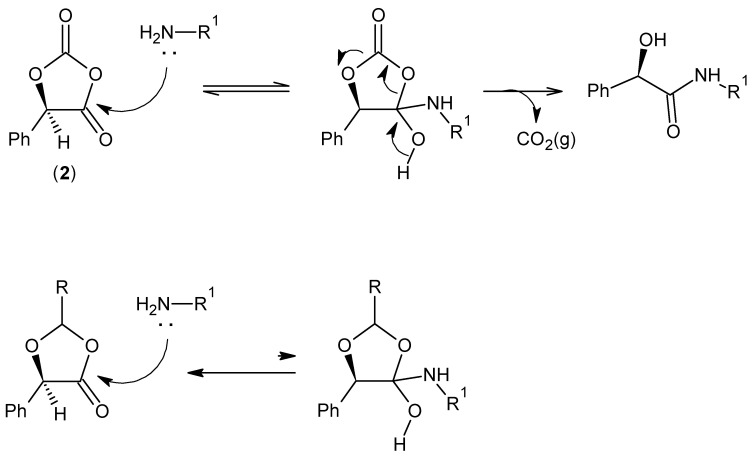
OCA decarboxylation.

Unfortunately, our efforts to obtain cefonicid using these molecules were in vain, even when we applied the same reaction conditions used for phosgene-derived OCA, having unsuccessfully studied amidation in water or in a mixture with polar aprotic solvents (acetone, ethyl acetate and acetonitrile [51]) at a neutral pH value and at weakly acidic and alkaline conditions, both at room temperature and in the 0–5 °C range. In addition, satisfactory results could not be achieved, despite the use of DMAP as an organic catalyst, such as in ring-opening polymerization (ROP) [43]. The reasons for failure lie in the fact that the irreversible decarboxylation reaction is the driving force in obtaining a new C-N bond, while formaldehyde and methyl/ethyl formate are bad leaving groups in nucleophilic substitutions, as reported in Figure 4. Finally, a line was drawn under these alternative compounds.

### 2.2. Amidation in Water and Organic Solvents

Sustainability is and will be the key driver and guideline in the future of API manufacturing, as will the goal of applying shorter synthetic routes that do not depend on hazardous reagents and solvents. It is now well established that amidation in an organic solvent is impossible for the extremely hydrophilic 7-SACA (**4**), and the same behavior was observed with environmentally friendly organic solvents such as 2-methyl-THF, cyclopentyl methyl ether (CPME), and cyrene. Only one protocol has been developed with harsh reaction conditions, and that was forty years ago. It was never industrialized because of the production of very dangerous waste, due to the use of *N*,*N*-dimethylformamide and 2,2′-dithiobisbenzothiazole [32]. By contrast, the amidation of the *O*-protected mandelic moiety in water has been the technique of choice on an industrial scale over the years because it combines high-grade drug quality with green chemistry credentials and the manufacturing requirements of good yields and low-cost production [52,53,54].

In the industry, both the ultrafiltration and reverse osmosis processes required to remove the enzyme from the aqueous medium and the 20 h needed for acid OH deprotection have seriously limited large-scale production because of the ultra-low productivity and heavy plant-management costs that these processes cause. Some years ago, Terreni et al. studied an enzymatic synthesis with immobilized acylase, which fell foul to a number of undesired, parallel reactions, low stability in the native protein, and irresolvable difficulty in recovering the biocatalyst from the reaction mixture, meaning that this approach was effectively abandoned [55].

Recent developments in the synthesis of the antimuscarinic agent, mirabegron, have prompted us to replicate the linkage of the mandelic group directly to 7-SACA (**4**) in an attempt to overcome the above-mentioned shortcomings. As amidation is one of the most commonly used chemical reactions in the synthesis of these semi-synthetic compounds, and as a broad class of coupling reagents exists, we focused our selection of activators on the practical aspects of standard chemistry processes: costs, yields, safety, and waste. As indicated in Table 1, activation via *O*-acylisoureic ester, boron-derived mixed anhydride, and sulfonate-based mixed anhydride are the techniques used in our work [56,57,58,59,60,61,62].

These data clearly highlight that it is impossible to directly attach (*R*)-mandelic acid (**1**) to 7-SACA (**4**) due to the presence of free OH, which interferes with the coupling reagent, meaning that the carboxylic moiety is not totally activated, and to the water medium used to dissolve the cephalosporanic nucleus, which hydrolyzes the activated adduct. When the same trials were repeated and the hydroxyl group was protected via formylation [30], it appeared that coupling via carbodiimide (EDC and DIC) promoted amidation in water, although less efficiently than the corresponding acyl chloride, generating formyl cefonicid, whereas the mixed anhydride was unsuccessful. The high reactivity of the acid chloride towards the 7-SACA (**4**) amine group led to a conversion of above 99% in solution towards formyl cefonicid, demonstrating that this reaction system possesses better adaptability in water, with the same results even being achieved when the OH-protected group was changed, as shown in Table 2.

These results placed us before an unwelcome choice because amidation via either EDC or DIC is explicitly more environmentally friendly than proceeding via acid chloride, due to the use of thionyl chloride. However, the difference in reactivity is so significant that acid chloride was chosen as the activation type.

### 2.3. Cefonicid Benzathine Salt

Our plan to develop a lean, cost-effective, and high-performance method for producing cefonicid has been achieved thanks to the simultaneous deprotection and crystallization of the drug with *N*′,*N*″-dibenzylethylenediamine diacetate (**5**) after the amidation step. This operation allows the ultrafiltration and reverse osmosis that are necessary to remove the enzyme from the reaction mixture in enzymatic deformylation to be avoided and makes the 20 h of reaction to deprotect the OH group with acid unnecessary.

**Scheme 1:**
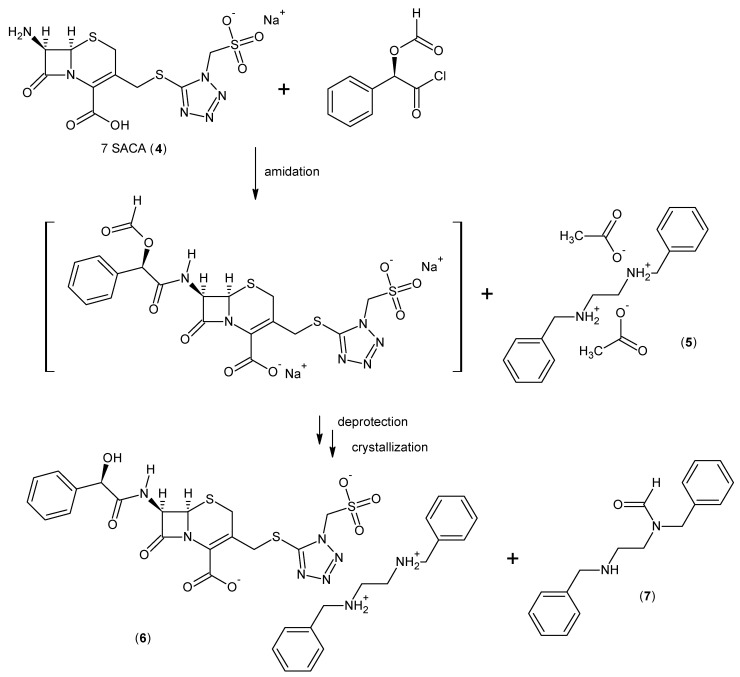
New protocol to obtain cefonicid benzathine salt (**6**).

Benzathine diacetate (**5**) is a common precipitating agent in cephalosporanic production thanks to its capacity to generate a salt between the cephalosporin negative charge and two secondary amines [54,63]. The linear chain with benzylic moieties at the bottom makes the molecule lipophilic in nature, enabling the precipitation of cephalosporin from the aqueous medium. In our case, we have taken advantage of the nucleophilicity of secondary amines to deprotect formyl cefonicid, and at the same time, crystallize the drug as a benzathine salt (**6**), Supplementary Figure S1, as described in Scheme 1.

This excellent formylic transfer surprisingly builds a new C-N bond on the reaction by-product, benzathine formylate (**7**), Figure S2, which is lost in the mother liquor after cake filtration, deblocks the hydroxy group on the cefonicid mandelic moiety, and all in the same step, is followed immediately by spontaneous product crystallization due to vigorous stirring. As reported in Table 3, the minimum amount of benzathine diacetate (**5**) required to achieve this excellent result is 2.4 equivalents, relative to 7-SACA (**4**), because the amidation/deformylation does not complete when working with lower amounts, while larger amounts do not affect the drug quality, thus meeting the theoretical assay for cefonicid (69.3%) and benzathine (30.7%).

We also investigated other amines (imidazole, pyridine, triethylamine, and 28% ammonia solution) in order to explore the reactivity of this OH-protected group, and we discovered that the same reactivity drove the formyl transfer but no crystallization occurred. These results represent a problem for our intent to streamline the process, as resins or chromatography would then be required to purify the product. However, at the same time, they also demonstrate that benzathine diacetate (**5**) shows the best performance of the amines tested.

Finally, we studied the same reaction on the OH-acetyl- and OH-pivaloyl-protected groups, but deprotection proved troublesome. In these cases, two subunits disconnected, with one being OH-acetyl-mandelic acid and the others being OH-pivaloyl-mandelic acid and 7-SACA (**4**).

As drug stability is a crucial feature when working on an industrial scale, we studied this parameter over 6 months and can confirm the high stability of the salt, as indicated in Table 4.

## 3. Materials and Methods

### 3.1. Chemistry

(*R*)-mandelic acid (**1**), *N*′,*N*″-dibenzylethane-1,2-diamine diacetate (**5**), *O*-acetyl-(*R*)-mandeloyl chloride, (*R*)-2-chloro-2-oxo-1-phenylethyl pivalate, *O*-formyl-(*R*)-mandeloyl chloride, and solvents were kindly provided by ACS Dobfar. All other reagents were purchased from Merck KGaA (Darmstadt, Germany).

The Agilent 1200 series HPLC system, NMR (Bruker 400, Munich, Germany Munich, Germany), and Karl Fischer titration were the analytical instruments used to identify and analyze the products.

#### 3.1.1. General Procedure for the Synthesis of *O*-Formyl-(*R*)-Mandelic Acid

A mixture of (*R*)-mandelic acid (**1**, 5.0 g, 32.9 mmol) and 99% formic acid (80 mL, 2.12 mol) was heated at 80–90 °C for 12 h and concentrated to give a residue. Toluene (100 mL) was added and evaporated under reduced pressure to remove formic acid as a binary azeotrope (86 °C at 101 KPa). The residual thick oil was dissolved in toluene (200 mL) and the solution was washed with water (2 × 50 mL). The organic layer was separated, dried over sodium sulphate (0.5 g), and evaporated under reduced pressure. The residual oil was taken up in cyclohexane (100 mL) to make the title compound a colorless solid (3.40 g, 57% yield), Figure S3. The crude ester was used directly for the next step without further purification.

#### 3.1.2. General Procedure for the Synthesis of 7-Amino-3-[sulphomethyl-1*H*-tetrazol-5-yl-thiomethyl]-3-cephem-4-carboxylate Monosodium Salt [7-SACA, **4**]

A solution of BF_3_ in MeCN (15.2–16.8% BF_3_ basis) (140 mL, 122.4 g, 288.8 mmol BF_3_) was added dropwise over 40 min to a stirred mixture of 1-sulphomethyl-5-mercapto-1,2,3,4-tetrazole sodium-potassium salt (7.7 g, 30.0 mmol) and 7-ACA (8.0 g, 29.3 mmol) in MeCN (60 mL), while the internal temperature was maintained at 25–28 °C. After 40 min under stirring, the reaction mixture was poured into the water (72 mL) at 25 °C, yielding a precipitate (Na, K borates). After filtration, the wet cake was washed with a (1:1) MeCN-water mixture (10 mL). The filtrates were pooled, and the precipitate was discarded. The limpid filtrate was brought to pH 3.0 ± 0.1 with 15% NaOH (45 mL) at 25 °C, and a precipitate became visible. This was aged for 2 h, filtered, and washed with acetone (32 mL) to make the title compound (**4**) (8.6 g, 68% yield) an off-white solid. The HPLC assay was 101.0% (as sodium on the anhydrous basis).

^1^H NMR (400 MHz, DMSO-d_6_): 3.50 (1H, d, ^2^*J* = 17.6 Hz), 3.74 (1H, d, ^2^*J* = 17.6 Hz), 4.08 (1H, d, ^2^*J* = 13.6 Hz), 4.42 (1H, d, ^2^*J* = 13.6 Hz), 5.20 (1H, d, ^3^*J* = 5.2 Hz), 5.40 (1H, d, ^3^*J* = 5.2 Hz), 5.87 (2H, s), Figure S6.

^13^C NMR (100 MHz, DMSO-d_6_): 26.25 (CH_2_), 36.0 (CH_2_), 53.3 (CH), 57.6 (CH), 60.4 (CH_2_), 119.8 (C), 130.0 (C), 154.9 (C), 159.6 (C), 161.8 (C), Figure S7.

#### 3.1.3. General Procedure for the Synthesis of Cefonicid Benzathine Salt (**6**)

A 30% (*w*/*w*) NaOH aqueous solution (24 mL) was added dropwise to a stirred suspension of 7-SACA (**4**, 100.0 g, 0.23 mol) in pre-cooled (0–5 °C) water (375 mL) until complete dissolution was achieved at pH 6.9–7.1. NaHCO_3_ (22.4 g, 0.26 mol) and O-formyl-(*R*)-mandeloyl chloride (52.0 g, 0.26 mol) were subsequently added to the reaction mixture, which was set aside at 0–5 °C for 45 min. The pH was adJ =usted to 5.8–6.1 via the addition of NaHCO_3_ (4.5 g, 0.053 mol) and alumina (2.5 g). After 20 min, the alumina was removed by filtration and washed with water (50 mL), and the filtrate was treated with norite charcoal (5 g) for 20 min at 20–25 °C. The charcoal was removed by filtration and the filtercake was washed with water (50 mL). Methanol (500 mL) was added, at 25–28 °C, to the combined filtrate, followed by 5% HCl (5 mL) to reach pH 5.1–5.2. *N*′,*N*″-dibenzylethylenediamine diacetate (**5**) (200.0 g, 0.55 mol) was added portionwise over 30 min. The reaction mixture was kept under fast stirring for 4 h to spontaneously crystallize the product. After stirring for an additional 5 h at 20–22 °C, water (600 mL) was added dropwise, and the resultant slurry was aged for 1 h. The solid was collected by filtration and subsequently washed with a (3:1) water–methanol mixture (2 L), followed by water (1700 mL) to make the cefonicid benzathine salt (**6**) (110.0 g; 63% yield) a colorless solid. M.p.: 195–198 °C; assay (HPLC, free base: 71.5% (theoretical maximum: 72.2%) for (1:1) cefonicid benzathine salt).

^1^H NMR (400 MHz, DMSO-d_6_): 8.76 (1H, d, *J* = 8.8 Hz), 8.0–7.0 (15H, m), 5.70 (1H, dd, *J* = 8.8 Hz, *J* = 4.8 Hz), 5.20–5.00 (4H, m), 4.27 and 4.23 (2H, AB system, *J* = 12.8 Hz), 4.11 (4H, s), 3.69 and 3.57 (2H, AB system, *J* = 17.6 Hz), 3.17 (4H, s), Figure S4.

^13^C NMR (100 MHz, DMSO-d_6_): 173.54 (CONH), 165.72 (CO β-lactam), 164.94 (COO), 156.01 (C-S), 141.46 (C), 138.58 (C-4), 130.86 (CH), 129.69 (C), 129.12 (CH), 129.00 (CH), 128.42 (CH), 127.96 (CH), 126.85 (CH), 121.21 (C), 121.8 (C-3), 73.52 (C-OH), 62.25(CH_2_SO_3_^−^), 58.99 (C-7), 57.95 (C-6), 51.54 (CH_2_-Ph), 44.60 (CH_2_N), 37.44 (CH_2_S), 27.32 (C-2), Figure S5.

## 4. Conclusions

In conclusion, we have reported a new, reliable, efficient, and sustainable protocol for the production of cefonicid benzathine salt (**6**) as both oral and injectable formulations. Using the double alkaline and nucleophilic behavior of *N*′,*N*″-dibenzylethylenediamine diacetate (**5**), we have considerably streamlined the process, producing the drug with remarkable stability and with a good yield, making it ready for industrial scale-up. The extreme reactivity of the OH-protected formyl group proved to be the winning weapon for this type of amidation, considering the bulkiness of benzathine diacetate (**5**), while its partially lipophilic nature facilitates the precipitation of the drug from the water medium and reduces waste production. In the jungle of API synthesis, where more and more companies are striving to find new ways to solve chemical-manufacturing challenges, we believe that this is a competitive process.

## Figures and Tables

**Table 1:**
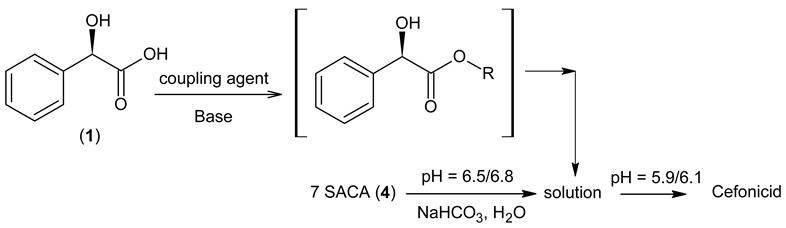
Direct amidation of (*R*)-mandelic acid (**1**).

Entry	Coupling Agent	Base	Solvent	Activation Temperature (°C)	Amidation Temperature (°C)	Cefonicid Yield in Solution (%)
1	TsCl	TEA	CH_3_CN	reflux	0–5 r.t.	0.5 1.3
2	MsCl	TEA	CH_3_CN	r.t.	0–5 r.t.	2.0 3.7
3	PivCl	TEA	CH_3_CN	0–5 °C	0–5 r.t.	- -
4	B(OMe)_3_	NaHCO_3_ ^a^	CH_3_CN	60 °C	0–5 r.t.	0.1 0.1
5	B(OMe)_3_	K_2_CO_3_ ^a^	CH_3_CN	60 °C	0–5 r.t.	0.1 0.1
6	EDC ^b^	-	water	r.t.	r.t.	1.2
7	DIC ^b^	-	water	r.t.	r.t.	0.3
8	TsCl	TEA	CH_2_Cl_2_^c^ EtOAc ^c^	reflux	r.t. r.t.	- -
9	MsCl	TEA	CH_2_Cl_2_^c^ EtOAc ^c^	r.t.	r.t. r.t.	0.1 1.0

Reaction conditions: 2.0 g of 7-SACA (**4**, 1.0 eq.), (*R*)-mandelic acid (**1**, 1.2 eq.), activating agent (1.2 eq. to (*R*)-mandelic acid), base (1.3 eq. to (*R*)-mandelic acid), water volume = 7.5 mL. ^a^ These entries use different bases to dissolve 7-SACA (**4**). NaHCO_3_ is the base for entry 4, while K_2_CO_3_ is the base for entry 5. ^b^ EDC and DIC were added as powders. ^c^ Solvent volume = 7.0 mL.

**Table 2:** Amidation with OH-protected mandelic acid.

Entry	OH-Protected Moiety	Coupling Agent	Solvent	OH-Protected Cefonicid Conversion in Solution (%) ^a^
1	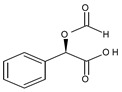	TsCl	CH_3_CN ^b^	1.4
2	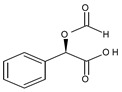	MsCl	CH_3_CN ^b^	4.0
3	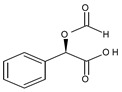	PivCl	CH_3_CN ^b^	0.5
4	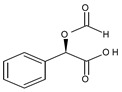	B(OMe)_3_	CH_3_CN ^b^	0.4
5	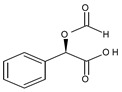	EDC	Water ^c^	27.0
6	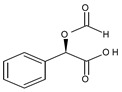	DIC	Water ^c^	32.0
7	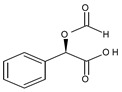	SOCl_2_	CH_3_CN ^d^	99.7
8	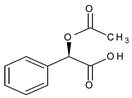	SOCl_2_	CH_3_CN ^d^	99.4
9	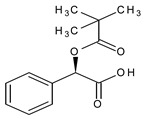	SOCl_2_	CH_3_CN ^d^	99.3

^a^ The formyl-cefonicid conversion is calculated as a peak-area ratio using the formula: ((OH-protected-cefonicid area)/(OH-protected-cefonicid area + 7-SACA (**4**) area)) × 100. ^b^ Reaction conditions: 2.0 g of 7-SACA (**4**, 1.0 eq.), *O*-formyl-(*R*)-mandelic acid (1.2 eq.), activating agent (1.2 eq. to *O*-formyl-(*R*)-mandelic acid), and base (1.3 eq. to *O*-formyl-(*R*)-mandelic acid). ^c^ Reaction conditions: 2.0 g of 7-SACA (**4**, 1.0 eq.), *O*-formyl-(*R*)-mandelic acid (1.2 eq.), and activating agent (1.2 eq. to *O*-formyl-(*R*)-mandelic acid). ^d^ Reaction conditions: 2.0 g of 7-SACA (**4**, 1.0 eq.) and OH-protected-(*R*)-mandelic acid (1.2 eq.) as a 2.8 mol/L concentration in CH_3_CN. The amidation step was always carried out at 0–5 °C.

**Table 3:** Reactivity study of formyl cefonicid with different *N*′,*N*″-dibenzylethylenediamine diacetate (**5**) amounts.

Entry	Equivalents ^a^	Conversion in Deformylation (%) ^b^	Crystallization ^c^	Yields (%)	Cefonicid Assay (%)	Benzathine Assay (%)
1	1.3	52	A	-	-	-
2	1.7	84	A	-	-	-
3	2.0	94	B	51	72.8	25.2
4	2.4	99.3	B	62	71.5	27.0
5	2.8	99.1	B	61	72.2	26.3

^a^ The equivalents were calculated as moles of benzathine diacetate (**5**)/mole of 7-SACA (**4**). ^b^ The cefonicid conversion is calculated as the peak-area ratio using the formula: ((cefonicid area)/(cefonicid area + formyl cefonicid area)) × 100. ^c^ A = no product crystallization, B = product crystallization.

**Table 4:** Salt stability over 6 months.

Time (Months)	Cefonicid Assay (%)	Benzathine Assay (%)	Total Impurities
0	71.5	27.0	1.9
6	71.6	26.8	1.7

## Data Availability

Data is contained within this article or Supplementary Materials.

## Supplementary Materials

The following are available online at https://www.mdpi.com/article/10.3390/antibiotics11081095/s1, Figure S1: IC analysis of cefonicid benzathine salt (**6**). Figure S2: Mother liquor LC-MS analysis to determinate benzathine formilate (**7**). Figure S3: *O*-Formyl-(*R*)-mandelic acid LC-MS analysis. Figure S4: ^1^H-NMR of cefonicid benzathine salt (**6**). Figure S5: ^13^C-NMR of cefonicid benzathine salt (**6**). Figure S6: ^1^H-NMR of 7-SACA (**4**). Figure S7: ^13^C-NMR of 7-SACA (**4**).
