# Supplementary material for: Cefonicid Benzathine Salt: A Convenient, Lean, and High-Performance Protocol to Make an Old Cephalosporin Shine
Source: Antibiotics (Basel). 2022 Aug 12;11(8):1095. doi: 10.3390/antibiotics11081095 (PMC9404797; doi:10.3390/antibiotics11081095)
Supplement: Supplementary file 1 [file antibiotics-11-01095-s001.zip › antibiotics-1843381-SI.pdf]

# Supplementary Materials

# Cefonicid Benzathine Salt: A Convenient, Lean, and High-Performance Protocol to Make an Old Cephalosporin Shine

Marziale Comito 1,2 , Riccardo Monguzzi 2, Silvia Tagliapietra 1 , Giovanni Palmisano 3

and Giancarlo Cravotto 1,*

1 Dipartimento di Scienza e Tecnologia del Farmaco, University of Turin, Via Pietro Giuria 9, 10125 Turin, Italy

2 Research and Development, ACS Dobfar SpA, Via Paullo 9, 20067 Tribiano, Italy

3 Dipartimento di Scienza e Alta Tecnologia, University of Insubria, Via Valleggio 9, 22100 Como, Italy

* Correspondence: giancarlo.cravotto@unito.it; Tel.: +39-011-6707183

## Figure captions

**Figure S1:** IC analysis of cefonicid benzathine salt (**6**).

**Figure S2**. Mother liquor LC-MS analysis to determinate benzathine formilate (**7**).

**Figure S3.** *O*-Formyl-(*R*)-mandelic acid LC-MS analysis.

**Figure S4.** $^{1}$H-NMR of cefonicid benzathine salt (**6**).

**Figure S5.** $^{13}$C-NMR of cefonicid benzathine salt (**6**).

**Figure S6.** $^{1}$H-NMR of 7-SACA (**4**).

**Figure S7.** $^{13}$C-NMR of 7-SACA (**4**).

**Figure S1**

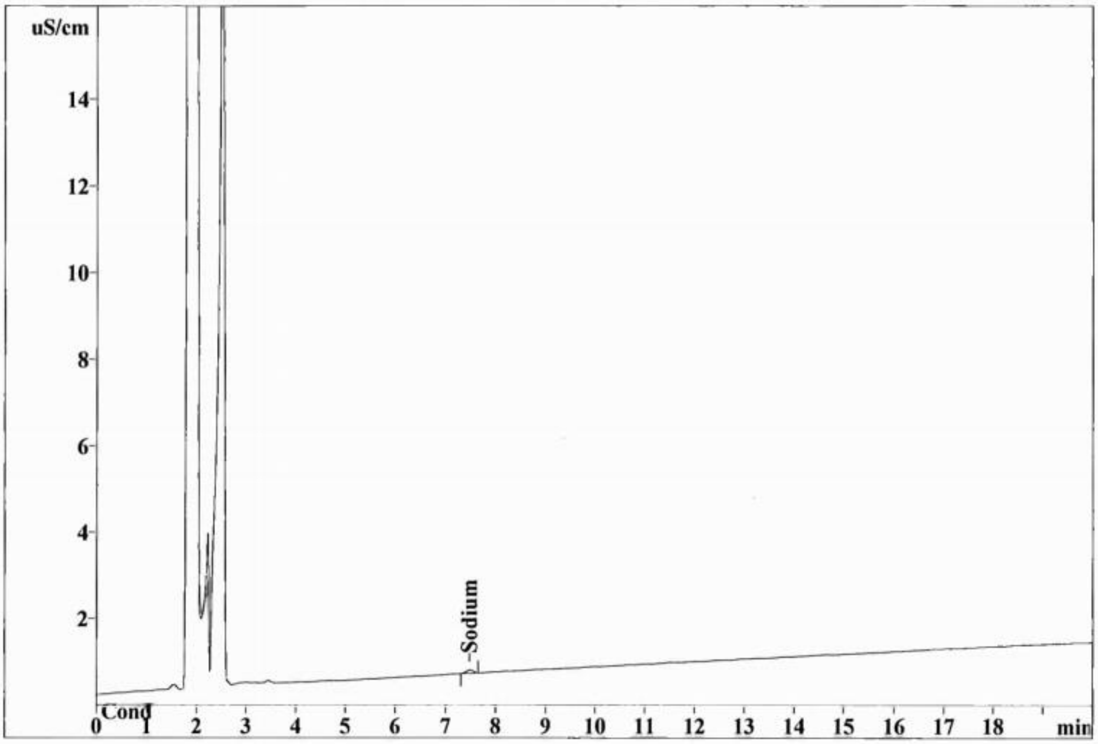


Quantitation method: Custom

| No | Retention min | Height uS/cm | Area uS/cm*sec | RF | Conc. mg/L | Name |
|---|---|---|---|---|---|---|
| 1 | 7.48 | 0.08 | 0.734 | 4.088 | 687.658 | Sodium |

**Figure S2**

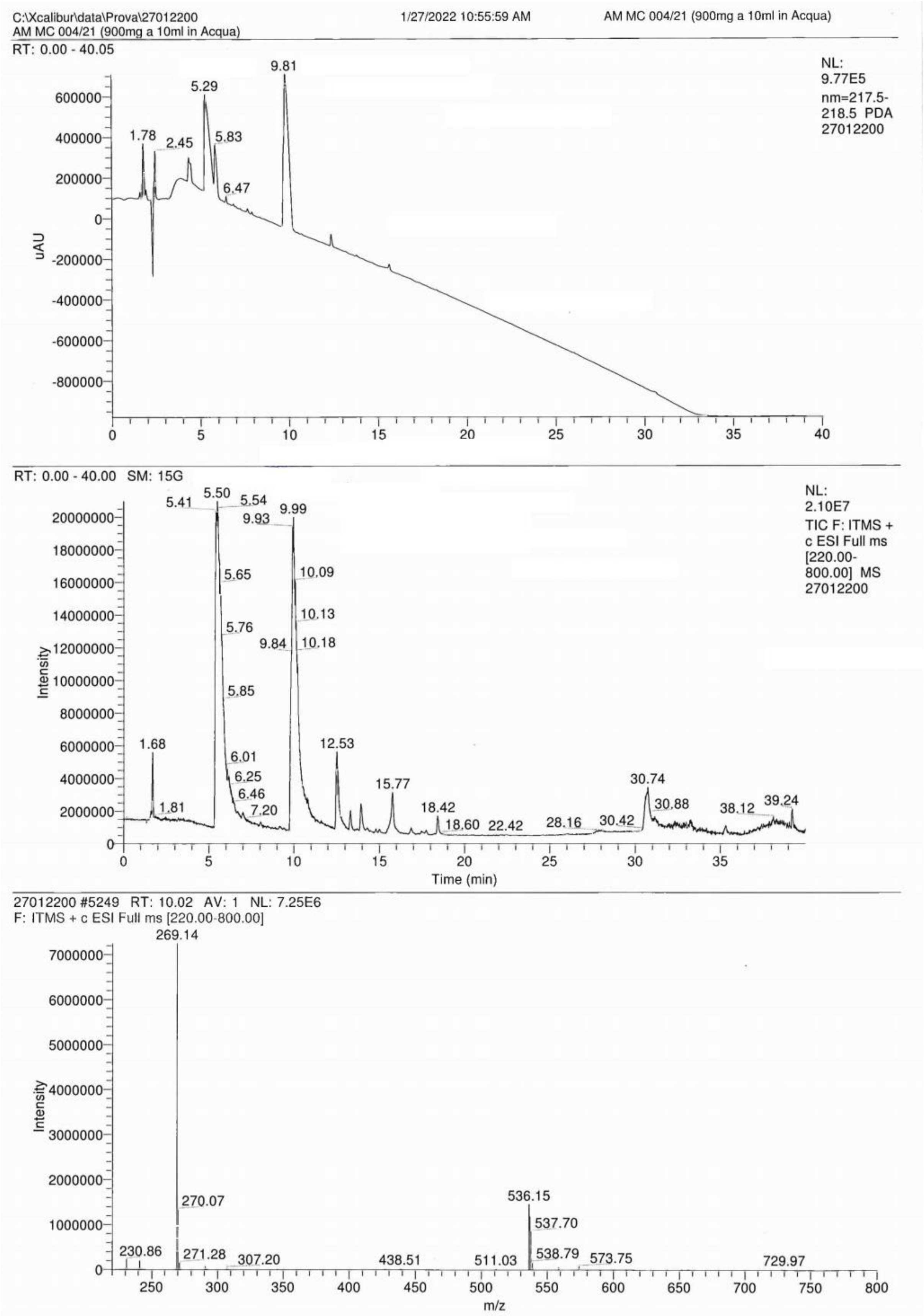

C:\Xcalibur\data\Prova\27012200
AM MC 004/21 (900mg a 10ml in Acqua)
1/27/2022 10:55:59 AM
AM MC 004/21 (900mg a 10ml in Acqua)
RT: 0.00 - 40.05
NL: 9.77E5 nm=217.5-218.5 PDA 27012200
uAU
RT: 0.00 - 40.00 SM: 15G
NL: 2.10E7 TIC F: ITMS + c ESI Full ms [220.00-800.00] MS 27012200
Intensity
Time (min)
27012200 #5249 RT: 10.02 AV: 1 NL: 7.25E6
F: ITMS + c ESI Full ms [220.00-800.00]
Intensity
m/z

**Figure S3**

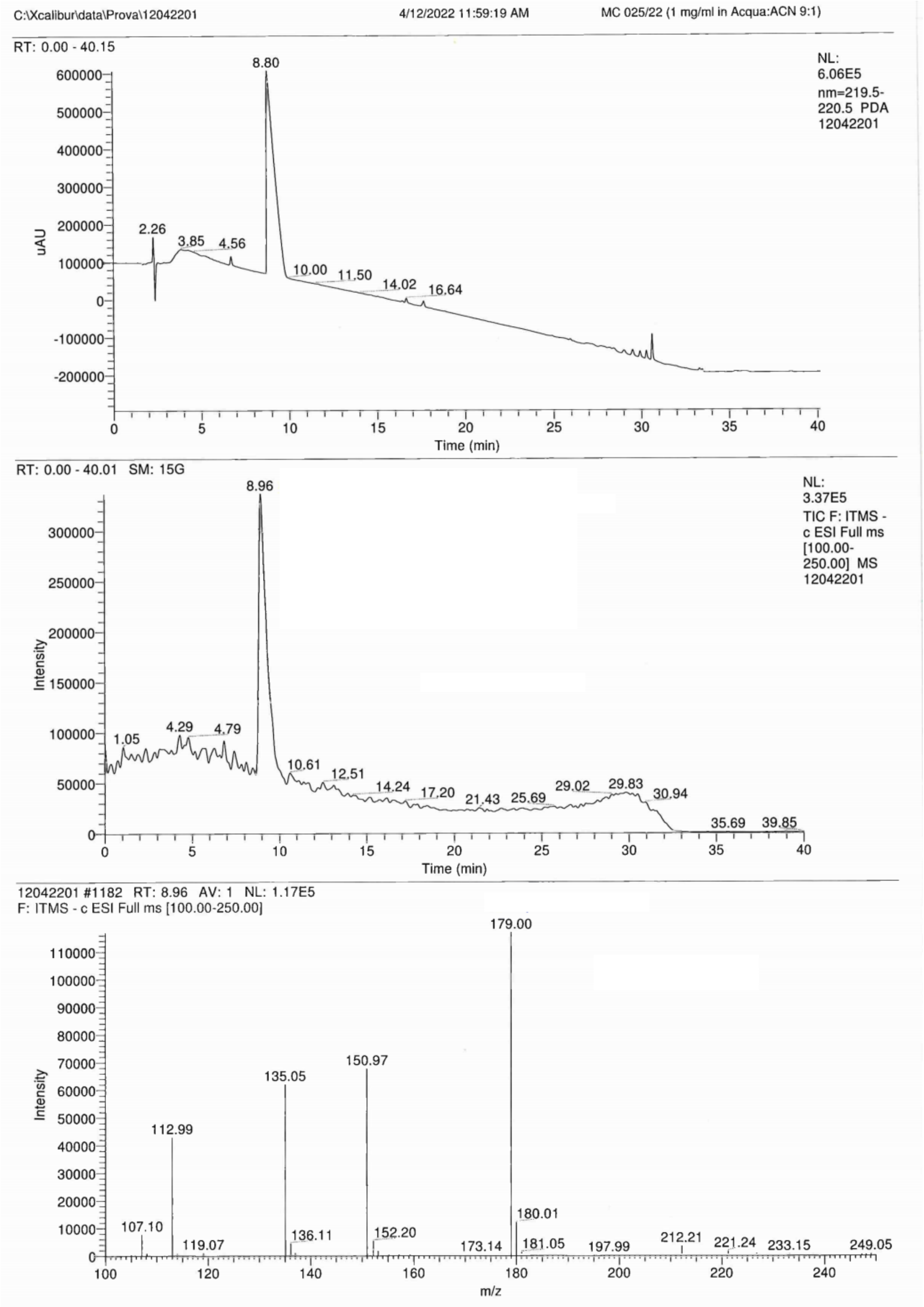
C:\Xcalibur\data\Prova\12042201
4/12/2022 11:59:19 AM
MC 025/22 (1 mg/ml in Acqua:ACN 9:1)
RT: 0.00 - 40.15
NL: 6.06E5 nm=219.5-220.5 PDA 12042201
uAU
Time (min)
8.80
2.26
3.85
4.56
10.00
11.50
14.02
16.64
RT: 0.00 - 40.01 SM: 15G
NL: 3.37E5 TIC F: ITMS - c ESI Full ms [100.00-250.00] MS 12042201
Intensity
Time (min)
8.96
1.05
4.29
4.79
10.61
12.51
14.24
17.20
21.43
25.69
29.02
29.83
30.94
35.69
39.85
12042201 #1182 RT: 8.96 AV: 1 NL: 1.17E5
F: ITMS - c ESI Full ms [100.00-250.00]
Intensity
m/z
179.00
150.97
135.05
112.99
107.10
119.07
136.11
152.20
173.14
180.01
181.05
197.99
212.21
221.24
233.15
249.05

**Figure S4**

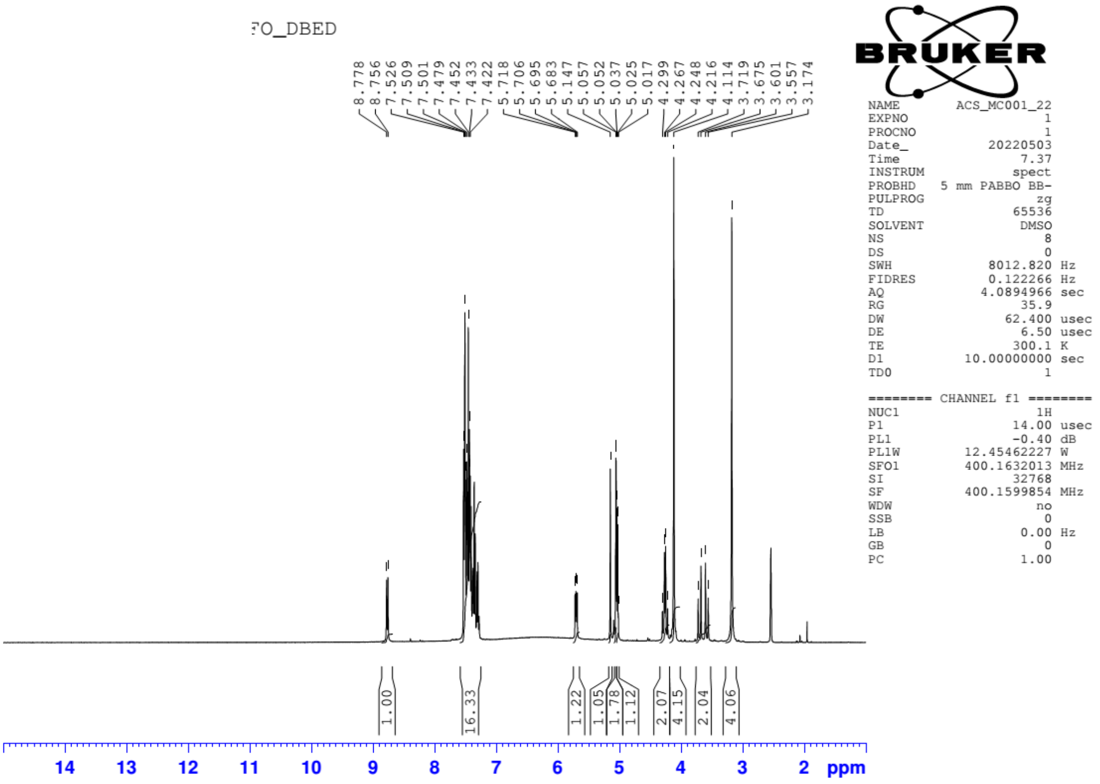
FO_DBED
8.778
8.756
7.526
7.509
7.501
7.479
7.452
7.433
7.422
5.718
5.706
5.695
5.683
5.147
5.057
5.052
5.037
5.025
5.017
4.299
4.267
4.248
4.216
4.114
3.719
3.675
3.601
3.557
3.174
NAME ACS_MC001_22
EXPNO 1
PROCNO 1
Date_ 20220503
Time 7.37
INSTRUM spect
PROBHD 5 mm PABBO BB-
PULPROG zg
TD 65536
SOLVENT DMSO
NS 8
DS 0
SWH 8012.820 Hz
FIDRES 0.122266 Hz
AQ 4.0894966 sec
RG 35.9
DW 62.400 usec
DE 6.50 usec
TE 300.1 K
D1 10.00000000 sec
TD0 1
======== CHANNEL f1 ========
NUC1 1H
P1 14.00 usec
PL1 -0.40 dB
PL1W 12.45462227 W
SFO1 400.1632013 MHz
SI 32768
SF 400.1599854 MHz
WDW no
SSB 0
LB 0.00 Hz
GB 0
PC 1.00
1.00
16.33
1.22
1.05
1.78
1.12
2.07
4.15
2.04
4.06
14
13
12
11
10
9
8
7
6
5
4
3
2
ppm

**Figure S5**

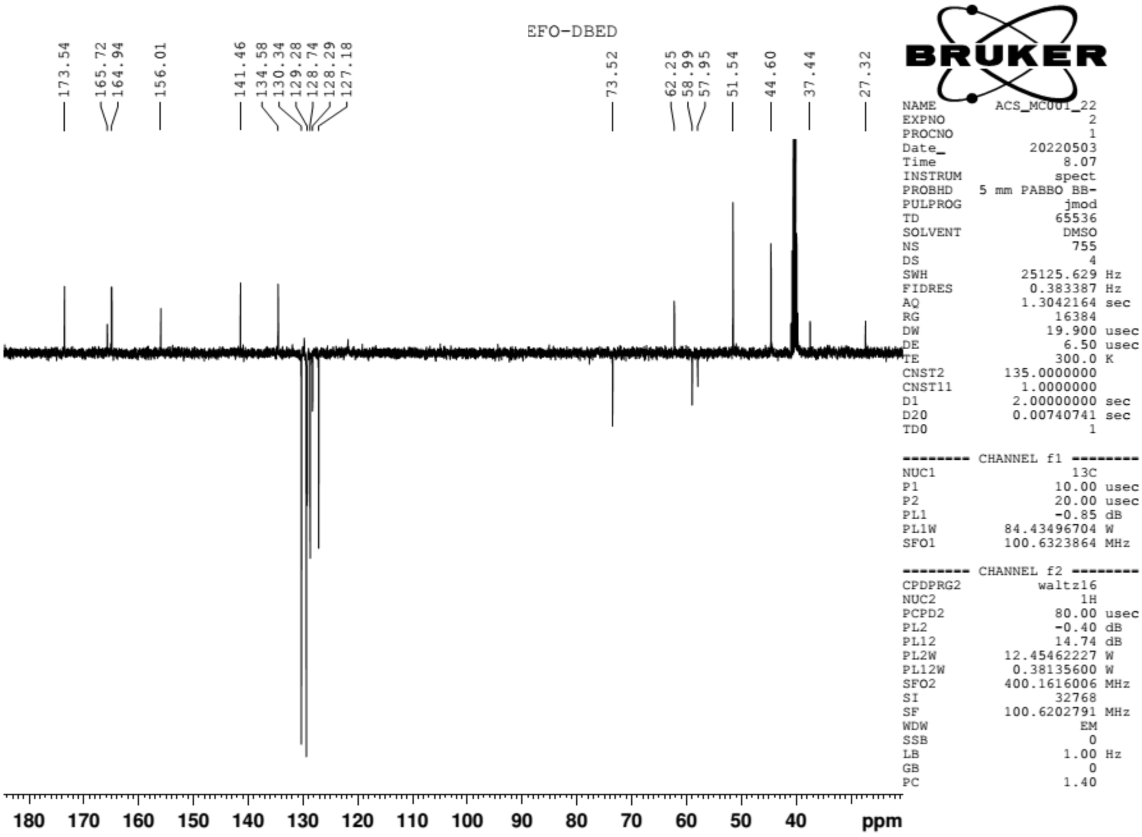
EFO-DBED
173.54
165.72
164.94
156.01
141.46
134.58
130.34
129.28
128.74
128.29
127.18
73.52
62.25
58.99
57.95
51.54
44.60
37.44
27.32
BRUKER
NAME ACS_MC001_22
EXPNO 2
PROCNO 1
Date_ 20220503
Time 8.07
INSTRUM spect
PROBHD 5 mm PABBO BB-
PULPROG jmod
TD 65536
SOLVENT DMSO
NS 755
DS 4
SWH 25125.629 Hz
FIDRES 0.383387 Hz
AQ 1.3042164 sec
RG 16384
DW 19.900 usec
DE 6.50 usec
TE 300.0 K
CNST2 135.0000000
CNST11 1.0000000
D1 2.00000000 sec
D20 0.00740741 sec
TD0 1
======== CHANNEL f1 ========
NUC1 13C
P1 10.00 usec
P2 20.00 usec
PL1 -0.85 dB
PL1W 84.43496704 W
SFO1 100.6323864 MHz
======== CHANNEL f2 ========
CPDPRG2 waltz16
NUC2 1H
PCPD2 80.00 usec
PL2 -0.40 dB
PL12 14.74 dB
PL2W 12.45462227 W
PL12W 0.38135600 W
SFO2 400.1616006 MHz
SI 32768
SF 100.6202791 MHz
WDW EM
SSB 0
LB 1.00 Hz
GB 0
PC 1.40
180 170 160 150 140 130 120 110 100 90 80 70 60 50 40 ppm

**Figure S6**

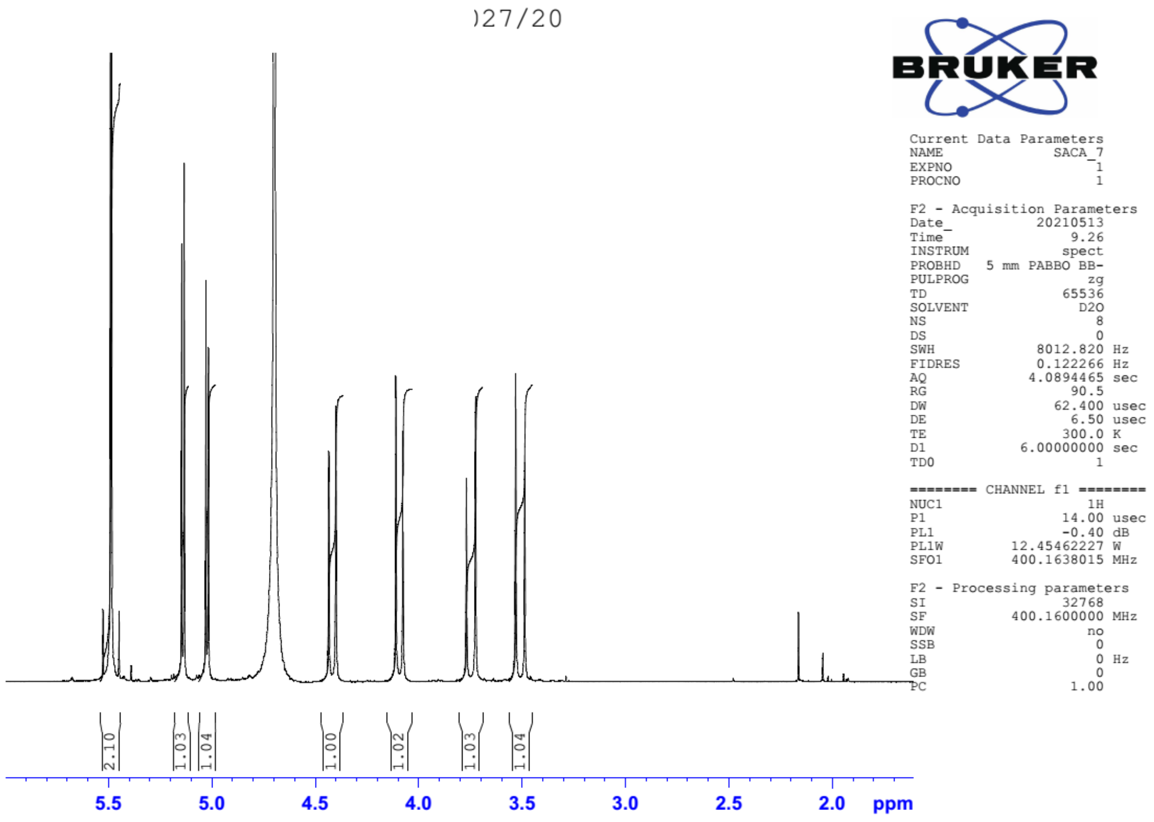
)27/20
BRUKER
Current Data Parameters
NAME SACA_7
EXPNO 1
PROCNO 1
F2 - Acquisition Parameters
Date_ 20210513
Time 9.26
INSTRUM spect
PROBHD 5 mm PABBO BB-
PULPROG zg
TD 65536
SOLVENT D2O
NS 8
DS 0
SWH 8012.820 Hz
FIDRES 0.122266 Hz
AQ 4.0894465 sec
RG 90.5
DW 62.400 usec
DE 6.50 usec
TE 300.0 K
D1 6.00000000 sec
TD0 1
======== CHANNEL f1 ========
NUC1 1H
P1 14.00 usec
PL1 -0.40 dB
PL1W 12.45462227 W
SFO1 400.1638015 MHz
F2 - Processing parameters
SI 32768
SF 400.1600000 MHz
WDW no
SSB 0
LB 0 Hz
GB 0
PC 1.00
2.10
1.03
1.04
1.00
1.02
1.03
1.04
5.5
5.0
4.5
4.0
3.5
3.0
2.5
2.0
ppm

**Figure S7**

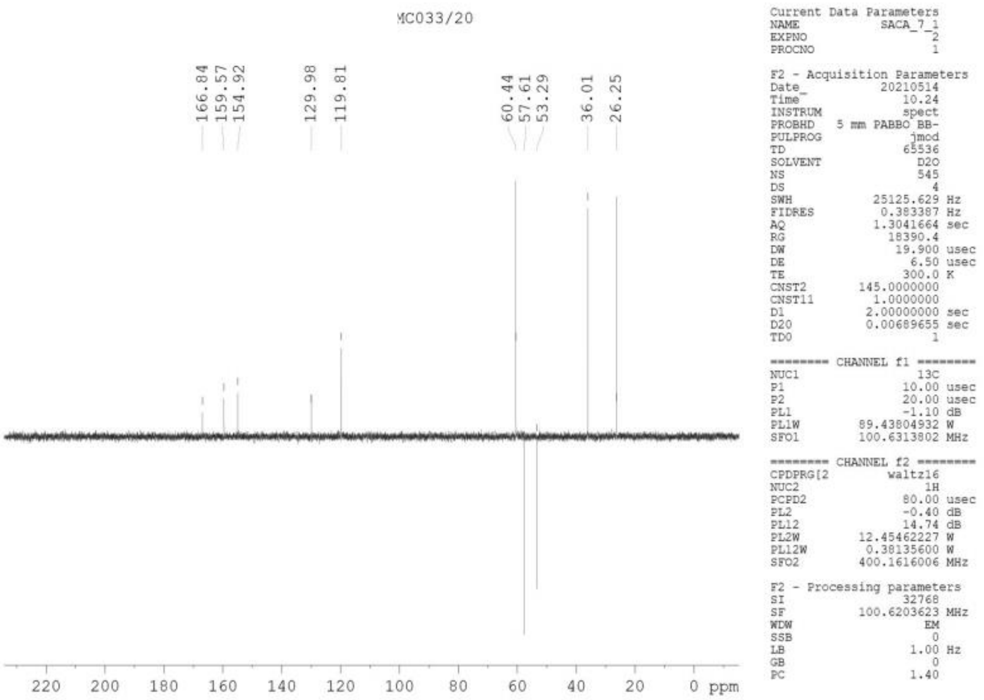
MC033/20
166.84
159.57
154.92
129.98
119.81
60.44
57.61
53.29
36.01
26.25
220 200 180 160 140 120 100 80 60 40 20 0 ppm
Current Data Parameters
NAME SACA_7_1
EXPNO 2
PROCNO 1
F2 - Acquisition Parameters
Date_ 20210514
Time 10.24
INSTRUM spect
PROBHD 5 mm PABBO BB-
PULPROG jmod
TD 65536
SOLVENT D2O
NS 545
DS 4
SWH 25125.629 Hz
FIDRES 0.383387 Hz
AQ 1.3041664 sec
RG 18390.4
DW 19.900 usec
DE 6.50 usec
TE 300.0 K
CNST2 145.0000000
CNST11 1.0000000
D1 2.00000000 sec
D20 0.00689655 sec
TD0 1
======== CHANNEL f1 ========
NUC1 13C
P1 10.00 usec
P2 20.00 usec
PL1 -1.10 dB
PL1W 89.43804932 W
SFO1 100.6313802 MHz
======== CHANNEL f2 ========
CPDPRG[2 waltz16
NUC2 1H
PCPD2 80.00 usec
PL2 -0.40 dB
PL12 14.74 dB
PL2W 12.45462227 W
PL12W 0.38135600 W
SFO2 400.1616006 MHz
F2 - Processing parameters
SI 32768
SF 100.6203623 MHz
WDW EM
SSB 0
LB 1.00 Hz
GB 0
PC 1.40
